# Completely Chiral Optical Force for Enantioseparation

**DOI:** 10.1038/srep36884

**Published:** 2016-11-09

**Authors:** Ivan D. Rukhlenko, Nikita V. Tepliakov, Anvar S. Baimuratov, Semen A. Andronaki, Yurii K. Gun’ko, Alexander V. Baranov, Anatoly V. Fedorov

**Affiliations:** 1 Center of Information Optical Technologies, ITMO University, Saint Petersburg 197101, Russia; 2 Monash University, Clayton Campus, Victoria 3800, Australia; 3 School of Chemistry and CRANN Institute, Trinity College, Dublin, Ireland

## Abstract

Fast and reliable separation of enantiomers of chiral nanoparticles requires elimination of all the forces that are independent of the nanoparticle handedness and creation of a sufficiently strong force that either pushes different enantiomers in opposite directions or delays the diffusion of one of them with respect to the other. Here we show how to construct such a completely chiral optical force using two counterpropagating circularly polarized plane waves of opposite helicities. We then explore capabilities of the related enantioseparation method by analytically solving the problem of the force-induced diffusion of chiral nanoparticles in a confined region, and reveal that it results in exponential spatial dependencies of the quantities measuring the purity of chiral substances. The proposed concept of a completely chiral optical force can potentially advance enantioseparation and enantiopurification techniques for all kinds of chiral nanoparticles that strongly interact with light.

Separation of opposite enantiomers of chiral drug molecules — which often have profoundly different pharmacokinetic, therapeuic, and toxicological properties — is one of the pressing problems that is slowing down the progress of pharmaceutical science and technology^1^. The task of enantioseparation is extremely challenging, because enantiomers are identical in most regards: they have the same densities and solubilities, boiling and melting points, electronic and vibrational frequencies, reactivities and refractivities, etc^2^. Only in their interactions with circularly polarized light, other chiral objects and substances do the two enantiomeric forms become distinguishable. This is true for all kinds of chiral nanoparticles, which are nowadays produced in great variety using the emerging fabrication techniques^3^,^4^,^5^. Among them are semiconductor nanocrystals with screw dislocations^6^,^7^ or chiral surfaces^8^,^9^,^10^,^11^,^12^,^13^. Much like enantiomeric molecules, chiral nanocrystals feature significantly different interactions with biomolecules and biological tissues^14^. For example, it has been experimentally demonstrated that certain cancer cells (e.g., HT1080 fibrosarcoma) exhibit a highly selective uptake of chiral CdS quantum dots (QDs), with an almost 100% uptake of the D-type QDs and a negligible uptake of the L-type QDs^15^. The need of individually testing each enantiomer of a chiral nanoparticle for its bioactivity and safety naturally extends the problem of enantioseparation from the pharmaceutical field to the entire field of chiral nanotechnology.

Although the classical methods for enantiomeric purity control based on various chromatographic techniques are quite efficient on analytical scale^16^,^17^, they still require significant optimisation to avoid the need of finding a suitable chiral selector using trial and error and to become highly efficient on an industrial scale. The same is relevant for the production of chiral drugs through asymmetric synthesis and separation of enantiomers by crystallisation^18^. Both processes result in chiral mixtures enriched with one enantiomer and requiring further purification. The existing purification techniques are quite expensive and inefficient at a large scale where kilograms of undesired enantiomers need to be removed from tens of kilograms of chiral drugs. These challenges, also faced by chiral nanotechnology, demand simple and cheap, yet unified and sensitive chiral analytical and separation methods. A number of original ways of separating enantiomers with the help of chiral light fields have been recently investigated as the possible basis for such methods^19^,^20^,^21^,^22^,^23^,^24^,^25^,^26^,^27^,^28^,^29^,^30^. In this paper we propose an original path to the unification of enantioseparation methods through the use of the simplest chiral object in optics — circularly polarised light. Our approach can potentially transform nanotechnology with regard to the resolution of chiral nanoparticle racemates.

## Results

There are two kinds of optical forces acting on a chiral nanoparticle exposed to an external electromagnetic field: an achiral force, whose direction is independent of the enantiomeric form of the nanoparticle, and a chiral one, whose directions are opposite for nanoparticles of different handednesses. The force of each kind has a reactive (i.e. conservative) component and a dissipative component. If a chiral nanoparticle, characterized by scalar electric, magnetic, and mixed electric–magnetic polarizabilities *α* = *α*′ + *iα*″, *β* = *β*′ + *iβ*″, and *χ* = *χ*′ + *iχ*″, interacts with a monochromatic field **E**(**r**, *t*) = Re(**E**_0_ *e*^−*iωt*^), then these four time-averaged forces are given by^26^


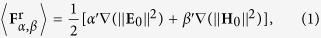



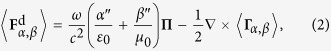



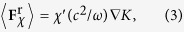



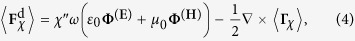
where 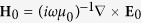, 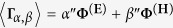 and 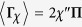 are the time-averaged torques acting on the nanoparticle, and where the Poynting vector, chirality density, and electric and magnetic ellipticities are defined as


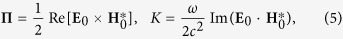



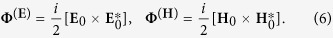


**Figure 1:**
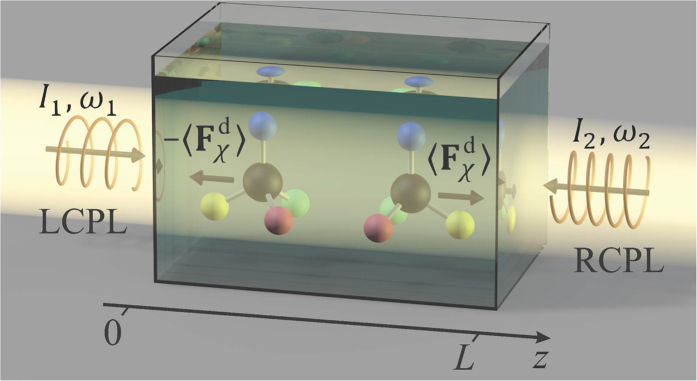
Separation of chiral nanoparticles with a totally chiral optical force exerted on them by two counterpropagating and noninterfering circularly polarized plane waves of the opposite handednesses.

Enantiomers of chiral nanoparticles can be separated optically only when they interact with chiral light fields. To enable enantioseparation in macroscopic volumes, one needs to avoid small-scale spatial variations of the fields’ intensities, and ensure that they exert on the nanoparticles only the chiral force while the achiral force is absent or negligibly small. It has been recently shown using the helicity momentum representation that the maximal chirality of a generic propagating electromagnetic field is achieved when the field is composed of plane waves of the same helicity^31^. It is reasonable therefore to begin constructing a completely chiral optical force by considering two counterpropagating circularly polarized plane waves of different frequencies, *ω*_1_≠*ω*_2_ (the difference of frequencies is needed to avoid interference between the waves and ensure the formation of a uniform chiral force). For such waves to produce a maximal chiral force they must have opposite helicities (handednesses), in which case their electric field can be represented in the form


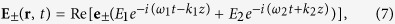
where *E*_*ν*_ and *k*_*ν*_ = *ω*_*ν*_/*c* are the amplitude and wave number of the *ν*th wave, and 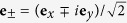 for the right circularly polarized wave propagating in the ±*z* direction. Figure 1 corresponds to field **E**_−_ (**r**, *t*) composed of left circularly polarized light (LCPL) travelling in the +*z* direction and right circularly polarized light (RCPL) travelling in the −*z* direction.

Owing to the absence of interference between the forward and backward waves, Eqs (1, 2, 3, 4, 5, 6) can be applied to each of them separately. This way we come to the conclusion that the intensity of the considered electromagnetic field is homogeneous, and the gradient force is absent, i.e. 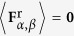. The chirality density of our field does not vary in space either and is given by the sum of the chirality densities of the two waves, i.e. *K*_±_ = *K*_1±_ + *K*_2±_, where *K*_*ν*±_ = ±(*k*_*ν*_/*c*)*I*_*ν*_, 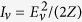 is the *ν*th wave intensity, and 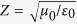 is the free-space impedance. This makes the chiral reactive force vanish too, 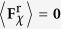.

The remaining two optical forces depend on the Poynting vector and the ellipticities of the electric and magnetic fields, which in our case are given by


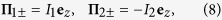



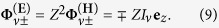


Note that while the Poynting vectors of the counterpropagating plane waves are oriented oppositely, the respective ellipticities point in the same direction due to the opposite helicities of the waves. Using these expressions in Eqs (2) and (4), we find


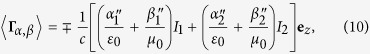



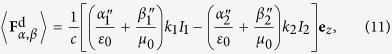



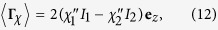



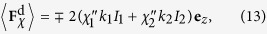
where the subscripts of polarizabilities mark the frequency at which they are taken and the upper or lower sign, as before, corresponds to the RCP wave moving in the positive or negative direction of the *z* axis. One can see that the achiral part of the dissipative force vanishes if the two terms in the square brackets of Eq. (11) cancel out. Taking into account that 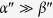, this condition can be written as


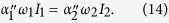


We have thus constructed a completely chiral, constant optical force, which enables separation of nanoparticle enantiomers on the macroscale. This force is accompanied by chiral and achiral torques, and can be enhanced by increasing the intensities of the two waves or using several pairs of counterpropagating plane waves, with intensities and frequencies in each pair matched according to Eq. (14). The raise of intensities is limited due to the radiation absorption by the nanoparticles and solution, which can lead to the decay of optical intensity and the associated force with the propagation distance, change the drift velocity of the enantiomers, or even destroy them and boil the solution at some point. In what follows we exclude such detrimental effects from consideration by assuming that the heat generation due to optical absorption is negligibly small. The total absorption rate by the nanoparticles is predominantly determined by the imaginary part of the electric polarizability and is given by


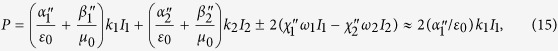
where we have used Eq. (14) and taken into account the hierarchy of polarizabilities, 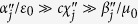. Of significance also is that 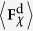 depends on the rotatory strengths *R*_*ba*_ of optical transitions occurring inside the nanoparticles, since 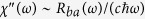
32. The chiral force reaches its maximum when *R*_*ba*_(*ω*_1_) and *R*_*ba*_(*ω*_2_) have the same sign. Polarizabilities 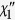 and 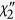 can be found from the circular dichroism (CD) spectrum, which is given by 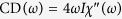 when it is measured with circularly polarized waves of intensity *I*.

All the expressions presented so far are written for electromagnetic fields in a vacuum. They can be generalized for a medium of permittivity *ε* and permeability *μ via* the standard replacements *ε*_0_ → *ε*_0_*ε*, *μ*_0_ → *μ*_0_*μ*, and 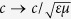.

**Figure 2:**
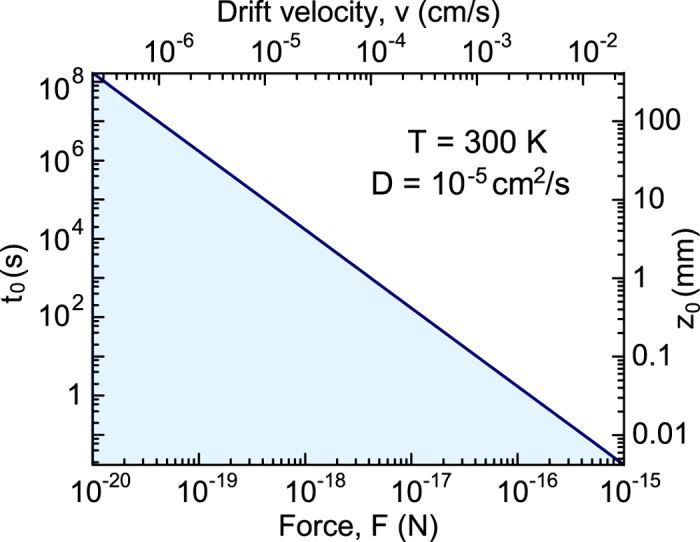
Characteristic time and length scales of spatiotemporal evolution of concentration of small molecules diffusing in water at room temperature in the presence of constant force *F*. The upper scale shows the respective drift velocity *v*.

We next study the force-induced diffusion of chiral nanoparticles by assuming that they are suspended in a solution and do not interact with each other. The diffusion of one enantiomeric form in a homogeneous force field 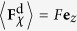 is governed by the Fokker–Planck equation


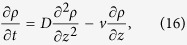
where *ρ*(*z*, *t*) is the enantiomer concentration, *D* is the diffusion coefficient, *v* = *FD*/(*k*_*B*_*T*) is the drift velocity of the nanoparticles under the driving force in the absence of diffusion, *k*_*B*_ is the Bolzmann constant, and *T* is the temperature of the solution. The diffusion equation is seen to contain two characteristic scales, *t*_0_ = *D*/*v*^2^ and *z*_0_ = *D*/*v*, which play the central role in the spatiotemporal evolution of the concentration. Figure 2 shows how these two scales vary with the force strength for a typical value of the diffusion coefficient of small molecules in water, *D* = 10^−5^ cm^2^/s.

Suppose that the solution is placed in a cuvette with impenetrable walls at *z* = 0 and *z* = *L* (see Fig. 1), and that the nanoparticles are initially distributed in it with a constant concentration *ρ*_0_. Then the conservation of the number of the nanoparticles requires their density mass flux to vanish at the walls. This leads us to the following initial and boundary conditions:


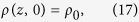



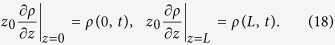


In order to explicitly take into account that the concentration of each enantiomer asymptotically approaches a certain stationary distribution — characterized by the zero density mass flux everywhere inside the cuvette — we use the ansatz


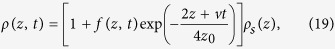
where 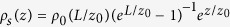 is the stationary solution to the diffusion equation. This ansatz reduces Eq. (16) to the diffusion equation of the standard form


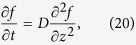
which is to be solved with the initial and boundary conditions


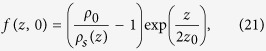



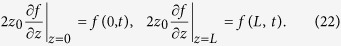


Note that in contrast to the concentration of the nanoparticles, which remains finite at all times, the unknown dimensionless function *f*(*z*, *t*) tends to zero as time grows indefinitely large.

The diffusion problem set up by Eqs (20, 21, 22) is now readily solved using the method of the separation of variables. We begin finding *f* by taking the general solution of Eq. (20) in the form 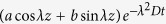. The condition at the left cuvette wall then relates the two constants of the spatial integration, *a* = 2*λz*_0_*b*, whereas the condition at the right wall determines the spectrum of *λ*: *λ*_*n*_ = *πn*/*L*, where *n* = 0, 1, 2, … As a result, we arrive at the family of solutions


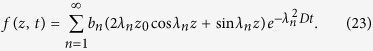


Finally, using the readily verifiable orthogonality of functions 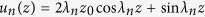 over the interval (0, *L*), from the initial condition we find


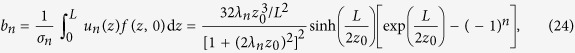
where


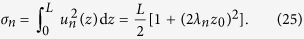


## Discussion

The obtained analytical solution gives us a clear picture of the spatiotemporal evolution of both enantiomers’ concentrations. First of all, Eqs (19) and (23) show that the effective constant of the temporal evolution can be approximated as


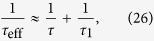
where *τ* = 4*t*_0_, 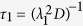, and *τ*/*τ*_1_ = (2*πz*_0_/*L*)^2^. If 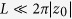, then 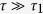 and the stationary distribution *ρ*_*s*_(*z*) is approached exponentially with a time constant *τ*_1_, which is independent of *F* and scales as ∝ *L*^2^/*D*. The opposite situation of 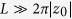 results in the steady state being reached over characteristic time *τ*, which is independent of *L* and scales as ∝ *T*/(*F*^2^*D*).

The purity of spatially separated enantiomers is characterised by the enantiomeric excess (*ee*) and enantiomeric ratio (*er*), which are given by the expressions


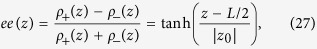



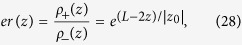
where *ρ*_+_(*z*) is the stationary concentration of the enantiomer that moves in the +*z* direction and *ρ*_−_(*z*) is the stationary concentration of the other enantiomer. Also informative are the integral measures of the spatial separation of enantiomers. We introduce three such measures as functions of a single dimensionless parameter *q* = *L*/*z*_0_:


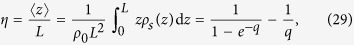



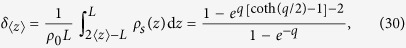



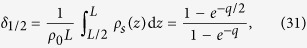
which are the normalized center of mass of the nanoparticles’ distribution, the relative number of the nanoparticles contained within the region of length 2(*L* − 〈*z*〉) about the center of mass, and the relative number of the nanoparticles in the right half of the cuvette. The last three definitions assume that the nanoparticles move to the right, i.e. *q* > 0.

**Figure 3:**
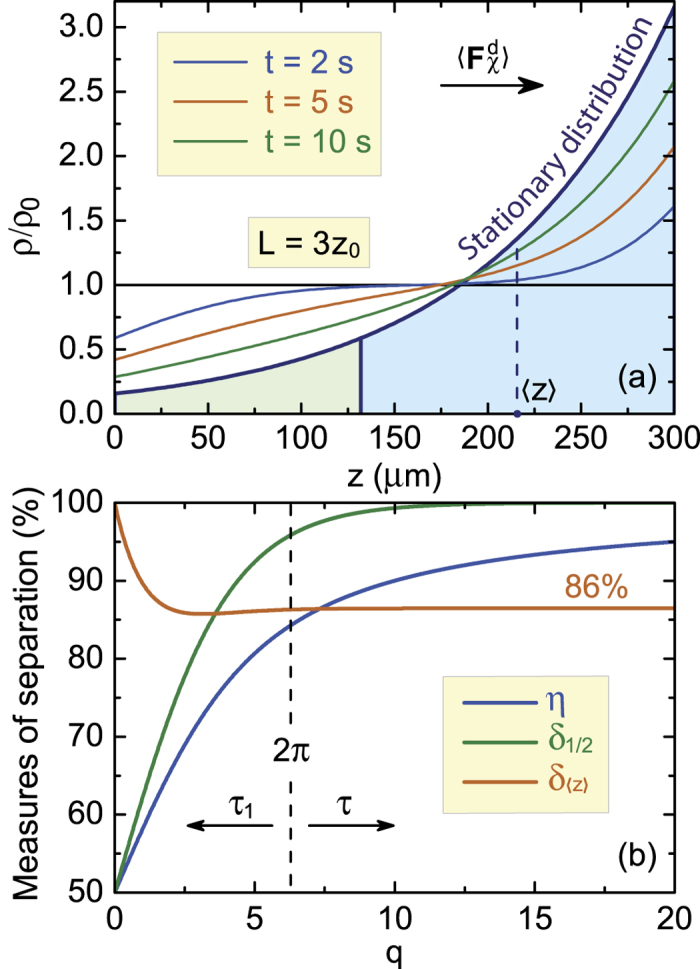
(**a**) Spatiotemporal evolution of concentration of enantiomers moved by a constant chiral force 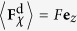, and (**b**) three integral measures of enantiomer separation. Shaded in blue is the area with the relative number of nanoparticles *δ*_〈*z*〉_. The temporal evolution is determined by *τ*_1_ for 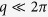 and by *τ* for 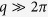. In (**a**) the simulation parameters are: *v* = 10^−3^ cm/s and *D* = 10^−5^ cm^2^/s.

The exponential dependencies of the quantities in Eqs (27, 28, 29, 30, 31) on the cuvette length and parameter *q* is a unique feature of enantioseparation with a completely chiral and constant optical force. This feature allows one to separate enantiomers to a degree of purity that is well beyond what is achievable with modern chromatography^16^,^17^. This fact is illustrated by Fig. 3. Let the enantiomers noticeably shift over time *t*_0_ = 10 s, which according to Fig. 2 corresponds to *z*_0_ = 0.1 mm and *v* = 10^−3^ cm/s, and requires a chiral force of about 4 × 10^−16^ N. Figure 3(a) shows how these parameters make the concentration of one enantiomer approach its stationary distribution in a cuvette of length *L* = 3*z*_0_. This length corresponds to *τ*_1_ ≈ 9 s, *τ*_eff_ ≈ 7 s, *η* ≈ 72%, *δ*_〈*z*〉_ ≈ 86%, and *δ*_1/2_ ≈ 82%, which are found from Eq. (26) and Fig. 3(b). In a larger cuvette, with *L* = 20*z*_0_, we have *τ*_1_ ≈ 405 s and *τ*_eff_ ≈ 36 s. The steady state is thus reached over a longer time, but the separation of enantiomers becomes almost complete, with *η* ≈ 95%, *δ*_〈*z*〉_ ≈ 86%, and *δ*_1/2_ ≈ 99.996%. Therefore, if a noticeable resolution of a racemate can be reached in a macroscopic volume over a reasonable time, then its complete resolution is also achievable over an acceptable time in a larger volume. We wish to reiterate that this is a unique feature of enantioseparation with a constant optical force and the direct consequence of the exponential dependencies given in Eqs (27, 28, 29, 30, 31).

The considered example shows that the complete enantioseparation on a submillimeter scale over times of a few hundreds of seconds requires chiral forces of the order of 10^−16^ N. Such forces are unattainable for small molecules, with typical rotatory strengths of about 10^−45^ J cm^3 ^33, but can be achieved at moderate optical powers for specifically designed chiral nanoparticles with high *χ*″. Indeed, if the molecules interact with 600-nm photons of energy 3.3 × 10^19^ J, then *cχ*″ ~ 3 × 10^−27^ cm^3^ and the light of intensity 1 MW/cm^2^ produces a chiral force of about 10^−24^ N. In order to achieve forces as high as 10^−16^ N with the same light intensity, one needs to have *χ*″ that is eight orders of magnitude larger. Such large values of *χ*″ can be featured by helix-type nanoobjects, including helix supercrystals^3^ and ‘Swiss-roll’ structures^34^. Note that although the proposed method is unsuitable for direct separation of chiral molecules, it can still be used to separate them if they are attached to chiral nanoparticles with large *χ*″.

As two concluding remarks, we would like to briefly discuss the issues of sample heating and extinction of light penetrating into the mixture. First of all, it is easy to make the heating of the system negligibly small by choosing the excitation wavelength in the transparency window of the colloidal solution and reducing the concentration of the nanoparticles. A simple heat balance equation shows that the temperature increase Δ*T* is inversely proportional to the mass *m* and heat capacity *C* of the solution and can be estimated as Δ*T* ~ *Pτ*_0_/(*Cm*), where *P* is the absorption rate given in Eq. (15) and *τ*_0_ is the characteristic heat dissipation time. By increasing the mass of the solution (or reducing the heat dissipation time) it is always possible to make the temperature of the system as stable as desired in practice. Second, one can estimate the impact of light extinction on the diffusion of the nanoparticles by assuming that the chiral force, which is proportional to the optical intensity, decays inside the cuvette exponentially with the propagation distance, *F*(*z*) = *F*_0_*e*^−*αz*^, where *α* is the absorption coefficient. The stationary solution to the diffusion equation in this case reads: 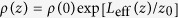, where *L*_eff_(*z*) = (1 − *e*^−*αz*^)/*α* is the effective coordinate. This expression shows that absorption converts exponential dependence of nanoparticle concentration on the coordinate to a saturable one. As a result, it reduces the effectiveness of enantioseparation the more the thicker the cuvette is and puts a fundamental limit on the extent of purity of spatially separated enantiomers achievable with our method. We intend to publish a detailed study of the role played by extinction in the diffusion process in our future paper elsewhere.

In conclusion, we have shown how to generate a completely chiral optical force by matching frequencies and intensities of two counterpropagating circularly polarized plane waves of opposite handednesses. We solved the problem of diffusion of chiral nanoparticles in the presence of such a force, and analyzed the peculiarities of the associated enantioseparation method. The method was shown to be capable of providing very high purities of chiral substances, beyond those achievable with modern chromatography.

## Methods

All graphs were plotted using Wolfram Mathematica 9.

## Additional Information

**How to cite this article**: Rukhlenko, I. D. *et al.* Completely Chiral Optical Force for Enantioseparation. *Sci. Rep.* **6**, 36884; doi: 10.1038/srep36884 (2016).

**Publisher’s note:** Springer Nature remains neutral with regard to jurisdictional claims in published maps and institutional affiliations.

## References

[b1] Crossley R. Chirality and the Biological Activity of Drugs (Boca Raton: CRC Press Inc., 1995).

[b2] Li B. & Haynie D. T. Chiral drug separation. *Encycl*. *Chem*. *Process*. 1, 449–458 (2006).

[b3] Baimuratov A. S., Gun’ko Y. K., Baranov A. V., Fedorov A. V. & Rukhlenko I. D. Chiral quantum supercrystals with total dissymetry of optical response. *Sci*. *Rep*. 6, 23321 (2016). 26991549 10.1038/srep23321 PMC4797577

[b4] Elliot S. D., Moloney M. P. & Gun’ko Y. K. Chiral shells and achiral cores in CdS quantum dots. *Nano Lett*. 8, 2452–2457 (2008). 18611059 10.1021/nl801453g

[b5] Zhang J., Albelda M. T., Liu Y. & Canary J. W. Chiral nanotechnology. Chirality 17, 404–420 (2005). 15991226 10.1002/chir.20178

[b6] Baimuratov A. S., Rukhlenko I. D., Gun’ko Y. K., Baranov A. V. & Fedorov A. V. Dislocation-induced chirality of semiconductor nanocrystals. *Nano Lett*. 15, 1710–1715 (2015). 25651415 10.1021/nl504369x

[b7] Baimuratov A. S. *et al.* Giant optical activity of quantum dots, rods, and disks with screw dislocations. *Sci*. *Rep*. 5, 14712 (2015). 26424498 10.1038/srep14712 PMC4589690

[b8] Rukhlenko I. D., Baimuratov A. S., Tepliakov N. V., Baranov A. V. & Fedorov A. V. Shape-induced optical activity of chiral nanocrystals. *Opt*. *Lett*. 41, 2438–2441 (2016). 27244383 10.1364/OL.41.002438

[b9] Tepliakov N. V., Baimuratov A. S., Baranov A. V., Fedorov A. V. & Rukhlenko I. D. Optical activity of chirally distorted nanocrystals. *J*. *Appl*. *Phys*. 119, 194302 (2016).

[b10] Purcell-Milton F., Govan J., Mukhina M. V. & Gun’ko Y. K. The chiral nano-world: Chiroptically active quantum nanostructures. *Nanoscale Horiz*. 1, 14–26 (2016). 10.1039/c5nh00072f 32260598

[b11] Mukhina M. V. *et al.* Intrinsic chirality of CdSe/ZnS quantum dots and quantum rods. *Nano Lett*. 15, 2844–2851 (2015). 25908405 10.1021/nl504439w

[b12] Yang B. *et al.* Dislocation-induced nanoparticle decoration on a GaN nanowire. *ACS Appl*. *Mater*. *Interfaces* 7, 2790–2796 (2015). 25562572 10.1021/am5079896

[b13] Moloney M. P., Govan J., Loudon A., Mukhina M. & Gun’ko Y. K. Preparation of chiral quantum dots. *Nature Protoc*. 10, 558–573 (2015). 25741991 10.1038/nprot.2015.028

[b14] Mukhina M. V. *et al.* Molecular recognition of biomolecules by chiral CdSe quantum dots. *Sci*. *Rep*. 6, 24177 (2016). 27063962 10.1038/srep24177 PMC4827062

[b15] Martynenko I. *et al.* Enantioselective cellular uptake of chiral semiconductor nanocrystals. Nanotechnology 27, 075102 (2016). 26782947 10.1088/0957-4484/27/7/075102

[b16] Gübitz G. & Schmid M. G. Chiral separation principles in chromatographic and electromigration techniques. *Mol*. *Biotechnol*. 32, 159–179 (2006). 16444017 10.1385/MB:32:2:159

[b17] Welch C. J. Chiral chromatography in support of pharmaceutical process research. In Cox G. B. (ed.) Preparative Enantioselective Chromatography (Blackwell Publishing Ltd, Oxford, UK, 2005).

[b18] Wang Y. & Chen A. M. Enantioenrichment by crystallization. *Org*. *Process Res*. *Dev*. 12, 282–290 (2008).

[b19] Hayat A., Mueller J. B. & Capasso F. Lateral chirality-sorting optical forces. *Proc*. *Natl*. *Acad*. *Sci*. *USA* 112, 13190–13194 (2015). 26453555 10.1073/pnas.1516704112 PMC4629360

[b20] Tkachenko G. & Brasselet E. Optofluidic sorting of material chirality by chiral light. *Nature Commun*. 5, 3577 (2014). 24717633 10.1038/ncomms4577

[b21] Tkachenko G. & Brasselet E. Helicity-dependent three-dimensional optical trapping of chiral microparticles. *Nature Commun*. 5, 4491 (2014). 25080171 10.1038/ncomms5491

[b22] Ding K., Ng J., Zhou L. & Chan C. T. Realization of optical pulling forces using chirality. *Phys*. *Rev*. *A* 89, 063825 (2014).

[b23] Donato M. G. *et al.* Polarization-dependent optomechanics mediated by chiral microresonators. *Nature Commun*. 5, 3656 (2014). 24710344 10.1038/ncomms4656

[b24] Cameron R. P., Barnett S. M. & Yao A. M. Discriminatory optical force for chiral molecules. *New J*. *Phys*. 16, 013020 (2014).

[b25] Bradshaw D. S. & Andrews D. L. Chiral discrimination in optical trapping and manipulation. *New J*. *Phys*. 16, 103021 (2014).

[b26] Canaguier-Durand A., Hutchison J. A., Genet C. & Ebbesen T. W. Mechanical separation of chiral dipoles by chiral light. *New J*. *Phys*. 15, 123037 (2013).

[b27] Nieto-Vesperinas M., Sáenz J., Gómez-Medina R. & Chantada L. Optical forces on small magnetodielectric particles. *Opt*. *Express* 18, 11428–11443 (2010). 20589003 10.1364/OE.18.011428

[b28] Tang Y. & Cohen A. E. Optical Chirality and Its Interaction with Matter. *Phys*. *Rev*. *Lett*. 104, 163901 (2010). 20482049 10.1103/PhysRevLett.104.163901

[b29] Li X. & Shapiro M. Communications: Spatial separation of enantiomers by coherent optical means. *J*. *Chem*. *Phys*. 132, 041101 (2010). 20113009 10.1063/1.3298585

[b30] Ma Y. & Salam A. On chiral selectivity of enantiomers using a circularly polarized pulsed laser under resonant and off-resonant conditions. *Chem*. *Phys*. 324, 367–375 (2006).

[b31] Bliokh K. Y. & Nori F. Characterizing optical chirality. *Phys*. *Rev*. *A* 83, 021803 (2011).

[b32] Choi J. S. & Cho M. Limitations of a superchiral field. *Phys*. *Rev*. *A* 86, 063834 (2012).

[b33] Tepliakov N. V. *et al.* Engineering optical activity of semiconductor nanocrystals *via* ion doping. Nanophotonics , 5, 517–522 2016).

[b34] Pendry J. B. A chiral route to negative refraction. Science 306, 1353–1355 (2004). 15550665 10.1126/science.1104467

